# Adsorption of Tetracycline with Reduced Graphene Oxide Decorated with MnFe_2_O_4_ Nanoparticles

**DOI:** 10.1186/s11671-018-2814-9

**Published:** 2018-12-05

**Authors:** Jian Bao, Yezi Zhu, Sijia Yuan, Fenghe Wang, Huang Tang, Zhihao Bao, Haiyun Zhou, Yajun Chen

**Affiliations:** 1 Jiangsu Province Key Laboratory of Environmental Engineering, Nanjing, 210036 China; 2 Jiangsu Provincial Academy of Environmental Science, Nanjing, 210036 China; 3 Jiangsu Provincial Key Laboratory of Materials Cycling and Pollution Control, School of Environment, Nanjing Normal University, Nanjing, 210023 China; 4 School of Mathematics and Physics, Jiangsu University of Technology, Changzhou, 213001 China; 5 Shanghai Key Laboratory of Special Artificial Microstructure Materials and Technology, School of Physics Science and Engineering, Tongji University, Shanghai, 200092 China

**Keywords:** Graphene oxide, Tetracycline, MnFe_2_O_4_, Adsorption

## Abstract

Nanomaterials were widely used as efficient adsorbents for environmental remediation of tetracycline pollution. However, the separation of the adsorbents posed the challenge to their practical applications. In this study, we grew magnetic MnFe_2_O_4_ nanoparticles on the reduced graphene oxide (rGO) to form MnFe_2_O_4_/rGO nanocomposite with a one-step method. When used as the absorbent of Tetracycline, it exhibited an adsorption capacity of 41 mg/g. The adsorption kinetics and isotherm were fitted well with the pseudo-second order model and Freundlich model, respectively. The MnFe_2_O_4_/rGO nanocomposite could be easily extracted from the solution with the external magnetic field and regenerated with acid washing.

**Electronic supplementary material:**

The online version of this article (10.1186/s11671-018-2814-9) contains supplementary material, which is available to authorized users.

## Introduction

Owing to its low toxicity with a broad spectrum of activity, tetracycline (TC) is one of the most widely used antibiotics in the world [1]. However, increasing concern has been raised in the recent years because TC is poorly degraded through metabolism. As a result, residual TC is directly discharged into the environment through feces and spread into nearby waterbodies and soil with water, causing the non-point pollution of those areas [1–3]. After the residue TC is accumulated in the human body, it exhibits chronic toxicity. Meanwhile, it can influence the aquatic photosynthetic organisms and indigenous microbial populations [4, 5]. To treat TC-polluted water, adsorption has been emerging as a promising method because it is efficient and cost-effective. The adsorbents used in adsorption include smectite clay [6], montmorillonite [7], diatomite [8], activated carbon [9], alumina [10], and carbon nanotube [11]. More recently, graphene-based nanomaterials has been used as the most effective adsorbents due to the existence of π-π interaction, H-bond, and cation-π bond between TC and graphene-based materials [12, 13]. Thus, these nanomaterials exhibit high adsorption capacities of TC. For example, theoretical maximum of adsorption capacity (*q*_*m*_) of graphene oxide and reduced graphene oxide can reach 313 and 558 mg/g, respectively [14, 15]. Graphene-based composite even exhibit higher adsorption capacities. TiO_2_/GO composite exhibits a *q*_*m*_ value of 1805 mg/g [16]. However, the separation of absorbents based on nanomaterials from polluted water poses a challenge to their practical applications. To facilitate the separation of the absorbent, magnetic absorbents were used. Our group demonstrated that thiol-functionalized magnetite/graphene oxide hybrid could be used as a reusable adsorbent for Hg^2+^ removal [17]. Chandra et al. utilized water-dispersible magnetite-reduced graphene oxide composites for arsenic removal [18]. In this study, we utilized Mn in the formation of GO to synthesize magnetic MnFe_2_O_4_/rGO composite with a one-pot method. MnFe_2_O_4_/rGO as the adsorbent exhibited relatively high adsorption capacity of 41 mg/g with an initial TC concentration of 10 mg/L. The magnetic adsorbent could be extracted from the water solutions easily with the help of the external magnetic field and reused after it was regenerated by soaking it in HCl aqueous solution.

## Materials and Methods

### Synthesis of GO

GO was prepared with a modified Hummer’s method. Briefly, H_2_SO_4_ (75.0 ml, 98 wt%) was slowly add in a flask with 1.0 g flake graphite and 0.75 g NaNO_3_ with mechanical stirring in an ice-water bath. After 10 min, 4.5 g KMnO_4_ was added gradually in the flask. With continuous and vigorous stirring, the mixture became pasty brownish, and then it was diluted with deionized water. H_2_O_2_ aqueous solution (20 ml, 30 wt%) was then slowly added into the mixture to form the GO mixture with Mn^2+^ ions.

### Synthesis of MnFe_2_O_4_/rGO Composite

We synthesized the MnFe_2_O_4_/rGO composite as reported previously [19]. Briefly, the above mixture was further diluted to 3000 ml with deionized water. FeCl_3_ (9.237 g) was dissolved in 400 ml deionized water, and then added into the mixture. Ammonia aqueous solution (30 wt%) was added to adjust its pH to 10 in 2 h. After the mixture was heated to 90 °C, hydrazine hydrate (98 wt%, 30 ml) was added slowly and stirred for 4 h, resulting in a black suspension. The suspension was cooled and was separated with magnets, washed with deionized water and ethanol several times, and finally dried in vacuum at 60 °C.

### Characterization of MnFe_2_O_4_/rGO Composite

X-ray diffraction (XRD) analysis was conducted with a diffractometer (Bruker D8 Discover) with Cu Kα radiation (40 kV, 40 mA). The morphology of samples was observed by a transmission electron microscope (TEM, JEOL 2100F). In this study, the vibrating sample magnetometer (VSM 7410, the Lake Shore) was used for the analysis of magnetic property of the nanocomposite.

### Determination of the Concentration of TC

**Fig. 1:**
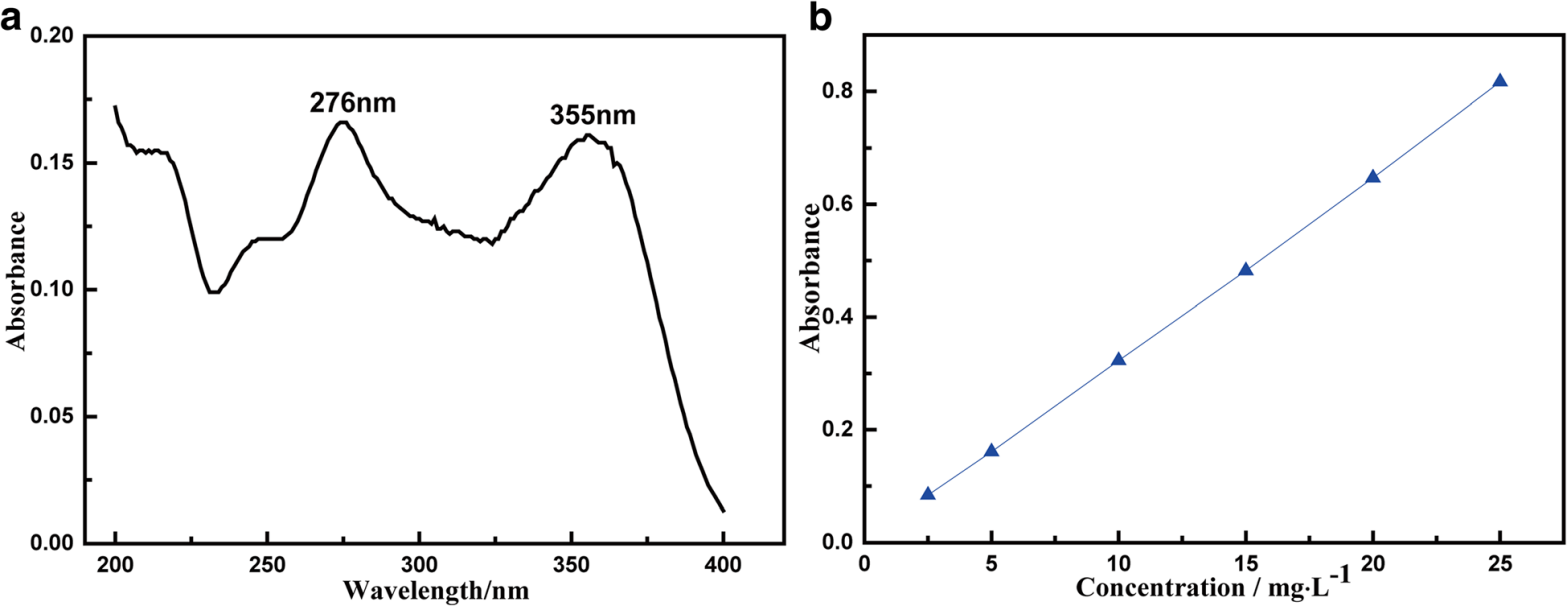
**a** UV spectrum and (**b**) calibrated curve for measurement of the concentration of TC

A thermostatic oscillator (ZD-85A) was used to ensure a steady and controllable adsorption process. An atomic absorption spectrophotometer (GTA 120, Agilent) was used to detect the ultraviolet characteristic absorption peak; and UV spectrophotometer (UV-1100, Shanghai mapada) was used to investigate the concentration of TC residue in solution by measuring the absorbance of the solutions. Other instruments involved in this study included pH meter (PHS-3C), drying oven (DHG-9240A), ultrasonic cleaner (KQ5200E), electronic scale (TP-214), and so on. TC (10 mg/L) solution was prepared for the linear calibration curve. Figure 1a showed the UV spectrum of TC. The characteristic adsorption peaks of are 276 nm and 355 nm. In this study, 355 nm was chosen as the scanning wavelength for TC adsorption. The calibration curve was presented in Fig. 1b. According to Lambert-Beer law [20], by measuring the absorbance of the solution, the concentration can be determined. The adsorption capacity (*Q*_*t*_, mg/g) and adsorption rate (*r*) are calculated by Eq. (1) and Eq. (2).

1 $$ {Q}_t=\frac{\left({C}_0-{C}_t\right)\times V}{m} $$

2 $$ \mathrm{r}=\frac{\left({\mathrm{C}}_0-{\mathrm{C}}_{\mathrm{t}}\right)}{{\mathrm{C}}_0}\times 100\% $$

where *C*_*0*_ (mg/L) and *C*_*t*_ (mg/L) are the concentration of TC residues in the solution in the beginning and at time *t,* respectively. V (mL) stands for the volume of the solution, and it is 30 mL in this study, and m (g) is the weight of the MnFe_2_O_4_/rGO sample used.

## Results and Discussion

### Synthesis and Characterization of MnFe_2_O_4_/rGO

**Fig. 2:**
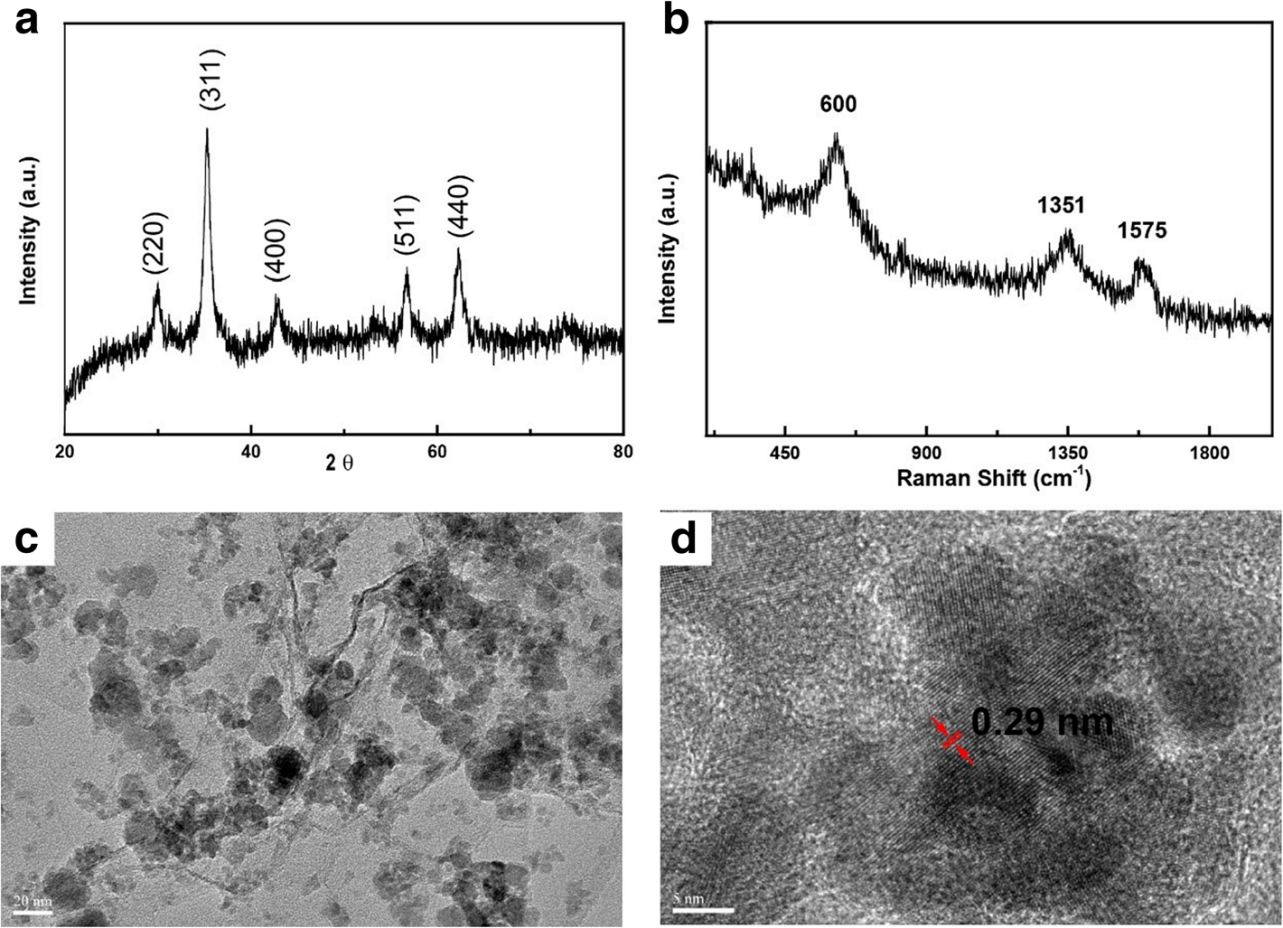
Characterization of the MnFe_2_O_4_/rGO nanocomposite. **a** XRD patterns and (**b**) Raman analysis of the nanocomposite; TEM image (**c**) and HRTEM image (**d**) of the nanocomposite

**Fig. 3:**
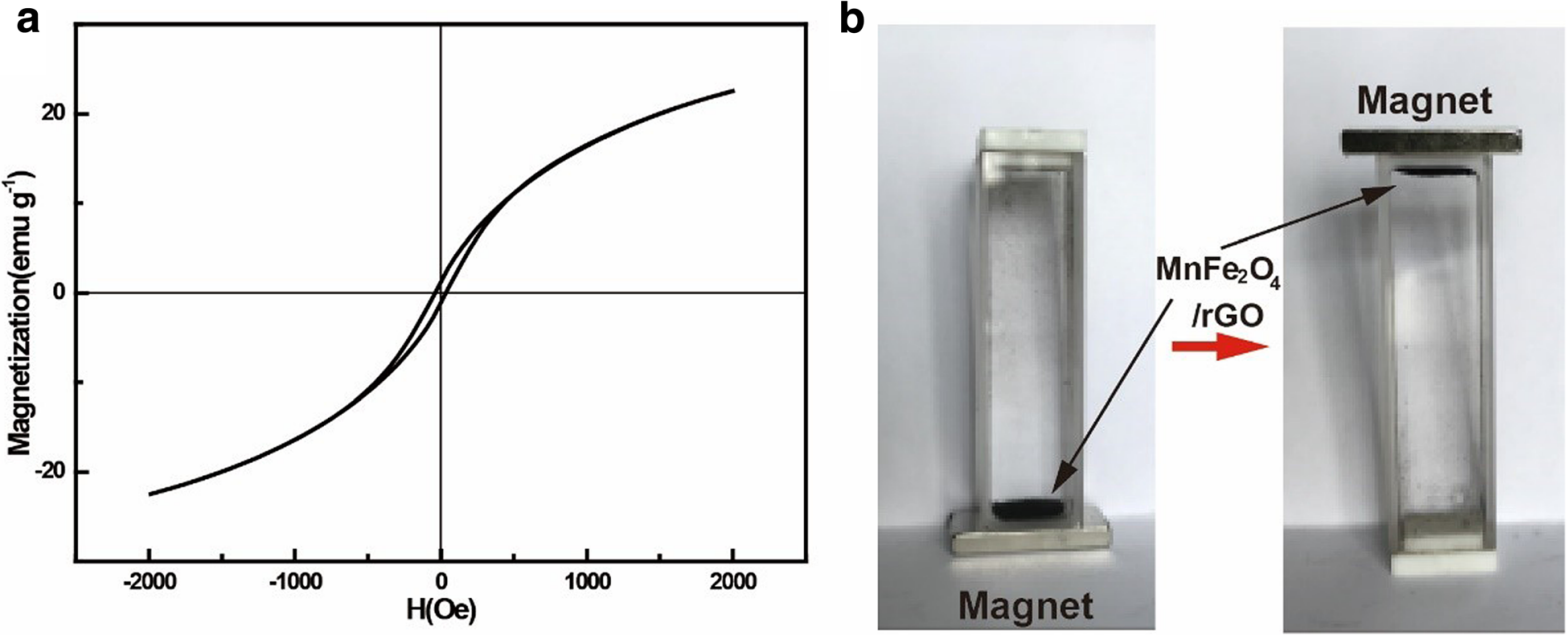
Magnetic property of the MnFe2O4/rGO nanocomposite. **a** Hysteresis loop and (**b**) magnetic separation of the nanocomposite from water

MnFe_2_O_4_/rGO nanocomposite was synthesized with a one-pot method as reported. In the process, we prepared a mixture containing GO with a modified Hummer’s method without purifying. Later, enough H_2_O_2_ aqueous solution was added in the mixture to reduce Mn ions with high valence to Mn^2+^ in the slurry. They were co-precipitated with Fe^3+^ in an alkaline environment to form MnFe_2_O_4_ nanocrystals on GO nanosheets which were reduced to graphene with the appearance of N_2_H_4_. MnFe_2_O_4_/rGO nanocomposite was finally formed. Figure 2a showed X-ray diffraction patterns of the nanocomposite. The diffraction peaks at 29.9, 35.5, 42.9, 56.8, and 62.3^o^ corresponded to the plane of (220), (311), (400), (511), and (440) of MnFe_2_O_4_ with the cubic phase (JCPDS card no. 10-319). In the Raman spectrum (Fig. 2b) of the composite, the peak at 600 cm^− 1^ was related to the vibration of MnFe_2_O_4_ while the other peaks at 1351 and 1575 cm^−1^ were D and G bands of rGO, respectively [21, 22]. The BET-specific surface area was 42.7 m^2^/g (Additional file 1: Figure S1). The high surface area was ascribed to the following reasons. During the synthesis process, GO nanosheets were used without purifying or drying. Meanwhile, MnFe_2_O_4_ nanoparticles nucleated and grew on them, keeping them from stacking. The weight ratios of rGO sheets and MnFe_2_O_4_ components in the MnFe_2_O_4_–rGO nanocomposite were evaluated to be approximately 12% and 88%, by thermal gravimetric analysis (Additional file 1: Figure S2) in air, respectively. TEM images (Fig. 2c) of the nanocomposite showed that MnFe_2_O_4_ nanoparticles with sizes below 30 nm were decorated on the nanosheets. High-resolution TEM images (Fig. 2d) of the nanocomposite further showed the clear lattice fringes with interplanar distances of 0.29 nm, corresponding to (220) planes of MnFe_2_O_4_ with cubic phase. The magnetic properties of the nanocomposite were examined with a magnetometer. A hysteresis loop of MnFe_2_O_4_/rGO at 25 °C was shown in Fig. 3a, the saturated magnetization and remanence magnetization were measured to be 22.6 emu/g and 1.1 emu/g, respectively. The small saturated magnetization was due to the small size of magnetite and appearance of GO in the composite. The coercivity of the nanocomposite was 39.0 Oe. The adsorbent with small remnant magnetization and coercivity at room temperature could be attracted and separated by even a small external magnetic field. In fact, MnFe_2_O_4_/rGO nanocomposite dispersed in water solution was easily extracted from water with a magnet, as confirmed by in optical image in Fig. 3b.

### Adsorption of TC on MnFe_2_O_4_/rGO

**Fig. 4:**
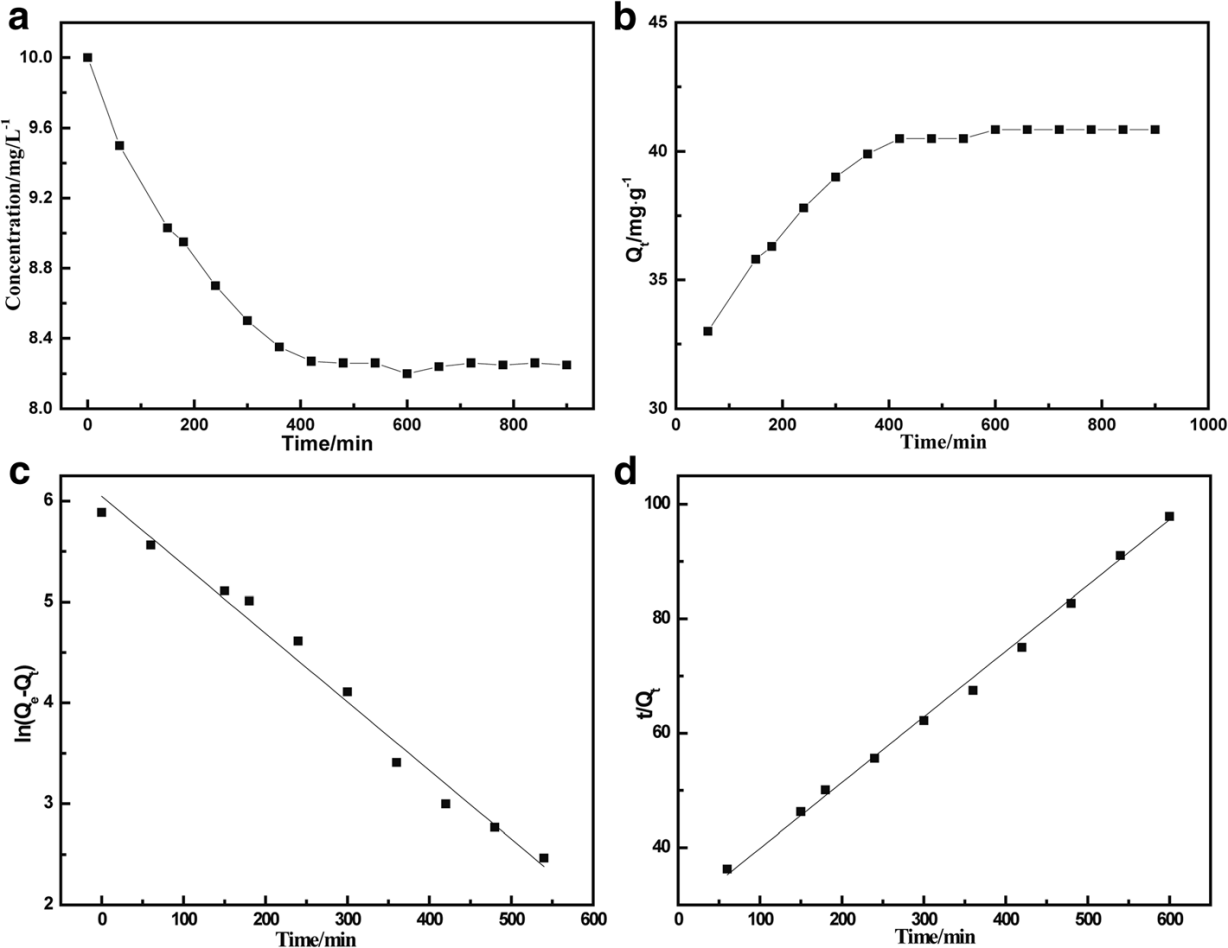
TC adsorption kinetics of MnFe_2_O_4_/rGO nanocomposite. **a** TC concentration and (**b**) adsorption capacity versus time during adsorption, and adsorption kinetics fitted with (**c**) pseudo-first-order kinetic model and (**d**) pseudo-second-order kinetic model

To investigate the adsorption kinetics, MnFe_2_O_4_/rGO (5 mg) was added into the TC solution (10 mg/L) at the temperature of 25 °C for adsorption. Then, the solution was placed in a temperature-constant oscillator to ensure sufficient mixing. Samples were taken at different times, and the absorbance of the sample was measured using the spectrophotometer. By comparing the calibration curve, the TC concentration in the solution at the different time during the adsorption process could be determined. Figure 4 showed the time influence on TC adsorption and the adsorption equilibrium, respectively. The adsorption process of TC on MnFe_2_O_4_ was moderately fast. It showed that the concentration of TC decreased dramatically during the first 5 h. Then, the adsorption process slowed down. After around 8 h adsorption, the concentration of TC solution was steady, implying the adsorption achieves equilibrium. The adsorption kinetics was slower than the pure GO dispersion [14], but faster than the magnetic graphene oxides sponge [23]. It is also much faster than adsorption of ciprofloxacin on the sodium alginate/GO. The adsorption kinetic might be related with the stacking structure of GO and how TC easily diffused to active adsorption site. According to Fig. 4b, the adsorption capacity was estimated be 41 mg/g with the initial TC concentration of 10 mg/L. This value was a little bit higher than that (39 mg/g) of GO-magnetic particles [24]. Two kinetic models, pseudo-first-order, and pseudo-second-order models, were applied here for the study of the adsorption mechanism. The pseudo-first-order dynamic equation is often used to simulate the solid-liquid adsorption system, with the linear expression shown in Eq. (3) [25]:

3 $$ \mathit{\ln}\left({q}_e-{q}_t\right)=\mathit{\ln}{q}_e-{K}_1t $$

where *q*_*e* (_mg/g) is the adsorption amount in equilibrium, and *q*_*t*_ (mg/g) is the amount of adsorption at time *t*. *K*_1_ is the rate constant of the pseudo-first-order kinetics. At the same time, the pseudo-second-order kinetics model is more widely applied to the adsorption kinetics of ions. The linear expression of the pseudo-secondary rate equation is shown in Eq. (4) [26]:

4 $$ \frac{t}{q_t}=\frac{1}{K_2{q}_e^2}+\frac{1}{q_e}t $$

Where *K*_2_ in this equation stands for rate constant of the pseudo-second-order kinetics.

Based on the experimental results of this study, Fig. 4c, d showed the fitting line of the adsorption by applying first-order adsorption kinetics and second-order adsorption kinetics, respectively. The detailed parameters of the two kinetics models are listed in Table 1.

**Table 1:** Kinetic models and related parameters used to fit the curves of adsorption

TC concentration (mg L^−1^)	First-order kinetics model		Second-order kinetics model	
	*K*_*1*_ × 10^−3^ (min^−1^)	*R* ^2^	*K*_*2*_ × 10^−3^ (g mg^−1^ min^−1^)	*R* ^2^
10	6.79	0.98282	114.87	0.99459

The correlation coefficient (*R*^*2*^, 0.99) for fitting of pseudo-second-order model was higher than that (0.98) of the pseudo-first-order model. It indicated that he pseudo-second-order kinetic model is suitable to describe the adsorption kinetics of TC on MnFe_2_O_4_/rGO nanocomposite. The kinetic constant *K*_2_ was 114.87 g mg min^−1^. To understand how TC interacted with MnFe_2_O_4_/rGO nanocomposite, Langmuir and Freundlich isotherm models were used to fit the adsorption data. Langmuir model is commonly expressed as Eq. (5) [27]:

5 $$ \frac{C_e}{q_e}=\frac{1}{K_L{q}_m}+\frac{C_e}{q_m} $$

where *C*_*e*_ (mg/L) is the equilibrium concentration, *q*_*e*_ (mg/g) is the amount of adsorption in equilibrium, *q*_*m*_ (mg/g) is the maximum monolayer adsorption capacity of adsorbent, *K*_*L*_, the Langmuir constant is related to the affinity between adsorbent and adsorbate. The values of *q*_*m*_ and *K*_*L*_ can be obtained by the slope of the equation and the intercept. Meanwhile Freundlich isotherm model is expressed as following equation [28]:

6 $$ \mathit{\ln}{q}_e=\mathit{\ln}{K}_F+\frac{1}{n}\mathit{\ln}{C}_e $$

where *K*_*F*_ is Freundlich constant and *n* is the adsorption index which describes the intensity.

**Fig. 5:**
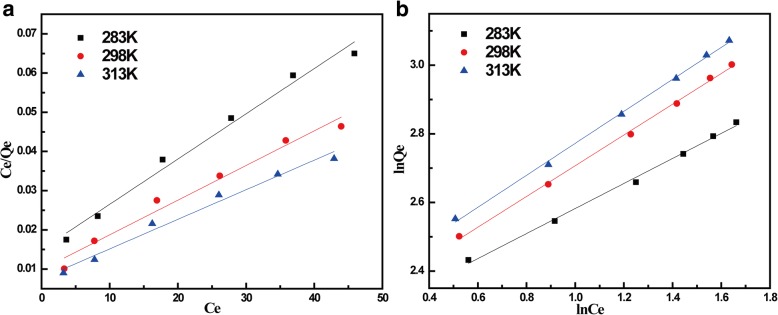
TC adsorption isotherms of MnFe_2_O_4_/rGO nanocomposite. Adsorption isotherms fitted with (**a**) Langmuir model and (**b**) Freundlich isotherm at 283, 298, and 313 K, respectively

To get an idea of the isotherm model of this kind of adsorption, the linear fitting using both Langmuir and Freundlich models are shown in Fig. 5, and the relevant parameters are listed in Table 2. As can be seen from Table 2, the adsorption of MnFe_2_O_4_/rGO to TC was fitted better with Freundlich isotherm than Langmuir isotherm. Freundlich adsorption model assumes that the adsorption is based on heterogeneous surface while Freundlich model is often used for non-ideal adsorption of different surfaces and multi-layer adsorption. The adsorption of tetracycline on rGO was related with the molecular structure of tetracycline and rGO. TC had four aromatic rings which could be easily adsorbed on rGO by the π-π interaction. Such interaction made multi-layer adsorption possible. It could attract additional TC molecules by the same interaction between TC molecules. The adsorption index *n* in this model was in the range of 2–3, which predicted that this adsorption system is “favorable.” When the temperature increased, the adsoportion capacity of TC on the nanocomposite also increased. It indicated that the adsorption process was endothermic.

**Table 2:** Adsorption isothermal parameters fitted with Freundlich and Langmuir models

Temperature	Freundlich			Langmuir		
	*K*_*F*_	*n*	*R* ^2^	*Q*_*m*_	*K*_*L*_	*R* ^2^
283	9.1812	2.7391	0.9951	870	0.0767	0.9838
298	9.5695	2.2304	0.9978	1131	0.0893	0.9723
313	10.0335	2.1443	0.9981	1326	0.0991	0.9795

**Fig. 6:**
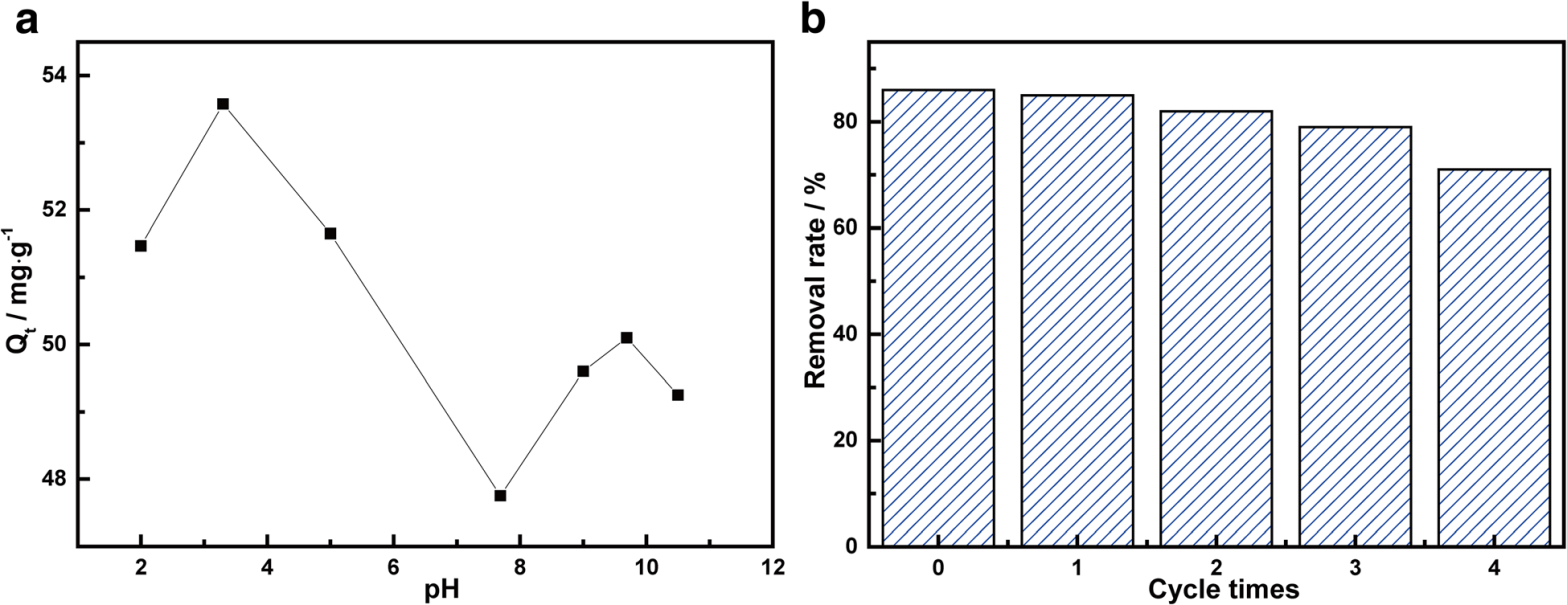
**a** Influence of pH on the adsorption of TC on MnFe_2_O_4/_rGO nanocomposite and (**b**) removal rate versus the cycling number with the initial TC concentration of 10 mg/L

To investigate the effects of pH on the adsorption, 30 mL TC solution (10 mg/L) and 5 mg of MnFe_2_O_4_/rGO powder were mixed, and solution pH was adjusted to 2.0, 3.3, 5.0, 7.7, 9.0, 9.7, and 10.5 at each test. The solution was placed in the oscillator at the temperature of 25 °C. Samples were taken at the adsorption equilibrium for measuring the concentration. Adsorption behavior under different pH was investigated, and the results tested under pH 2.0 to 10.5 were shown in Fig. 6. The maximum adsorption capacity of MnFe_2_O_4_/rGO in TC takes place when solution pH was 3.3. When pH was less than 3.3, the adsorption decreased with the increase of acidity. This was mainly because of the competition on the adsorption sites between TCH^3+^ and large amounts of H^+^ ions in the solution. When pH was between 3.3 and 7.7, the TC existed in the form of TCH_2_^0^. The electrostatic interaction was week. With the solution became more alkaline, the increased OH^−^ might cause sedimentation with the metallic ion from MnFe_2_O_4_/rGO and thus reduce the adsorption. At pH = 9.7, this was exactly the transition point where the dominant TC formed in the solution changes from TCH^−^ to TC^2−^. Thus, it is assumed that the existence of the peak at pH = 9.7 was due to change of ion forms in the solution. In this study, HCl solution (0.1 mol/L) was used as an eluent to find out the adsorption-regeneration characteristics of MnFe_2_O_4_/rGO to TC. Adsorption was carried out at 25 °C, with 5 mg of MnFe_2_O_4_/rGO adding into the 10 mg/L TC solution. After adsorption equilibrium, MnFe_2_O_4_/rGO was eluted by HCl solution. Then, the eluted MnFe_2_O_4_/rGO was used for adsorption again, and the adsorption capacity was measured. The elution was carried out for three times, and by comparing the adsorption capacity after each elution, the adsorption-regeneration characteristic was drawn. In this study, all the tests were run at least three times. The oscillator in all the experiments was set to a fixed speed of 180 rpm. Figure 6b showed the adsorption-regeneration behavior of MnFe_2_O_4_/rGO on TC adsorption. The initial removal rate was 86%. After being eluted by HCl, the removal rate of TC was 85%, 82%, 79%, and 71% for the first 4 cycles. It indicated that the adsorbents could be easily regenerated and reused.

Overall, we believed that rGO mainly contributed the adsorption of TC. Firstly, the size of MnFe_2_O_4_ reached several tens of nanometers; it could not contribute a lot to the overall surface area. Secondly, the overall adsorption capacity was ~ 40 mg/g in TC with an initial concentration of ~ 10 mg/mL. This value was almost the same with the reported adsorption capacities of GO [14]. The appearance of magnetic MnFe_2_O_4_ made the extraction and recycling of the adsorbent, rGO, easily.

## Conclusions

MnFe_2_O_4_/rGO nanocomposite was successfully synthesized with one-pot method. The nanocomposite could be used as efficient adsorbents of TC with the adsorption capacity of 41 mg/g when the initial TC concentration was 10 mg/L. The kinetics and isotherm of the adsorption process was described as the pseudo-second-order model and Freundlich model, respectively. The magnetic adsorbents can be separated and regenerated, indicating the MnFe_2_O_4_/rGO nanocomposite can be a promising reusable adsorbents for environmental remediation for TC pollution.

## Additional file

Additional file 1: **Figure S1.** Nitrogen adsorption-desorption isotherms of MnFe_2_O_4_-rGO. **Figure S2.** Thermogravimetric analyses of MnFe_2_O_4_-rGO in air. (DOCX 220 kb)

## Abbreviations

GO: Graphene oxide
rGO: Reduced graphene oxide
TC: Tetracycline
TEM: Transmission electron microscopy
