# Supplementary material for: Adsorption of Tetracycline with Reduced Graphene Oxide Decorated with MnFe2O4 Nanoparticles
Source: Nanoscale Res Lett. 2018 Dec 5;13:396. doi: 10.1186/s11671-018-2814-9 (PMC6281545; doi:10.1186/s11671-018-2814-9)
Supplement: Supplementary file 1 — Figure S1. Nitrogen adsorption-desorption isotherms of MnFe2O4-rGO. Figure S2. Thermogravimetric analyses of MnFe2O4-rGO in air. (DOCX 220 kb) [file 11671_2018_2814_MOESM1_ESM.docx]

Additional file

Adsorption of Tetracycline with reduced graphene oxide decorated with MnFe_2_O_4_ nanoparticles

Jian Bao, Yezi Zhu, Sijia Yuan, Fenghe Wang*, Huang Tang*, Zhihao Bao, Haiyun Zhou, Yajun Chen


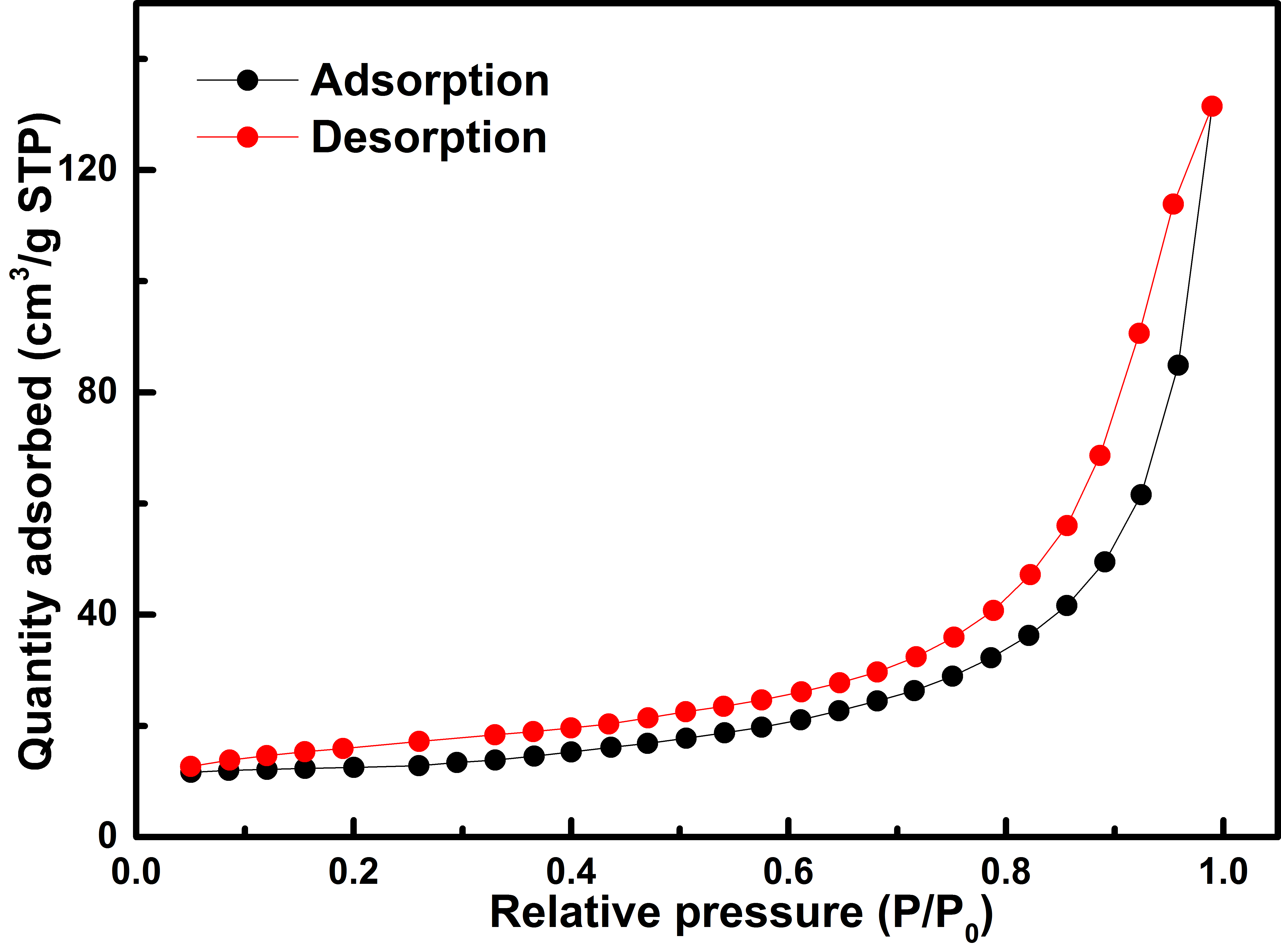


Figure S1: Nitrogen adsorption-desorption isotherms of MnFe_2_O_4_-rGO.


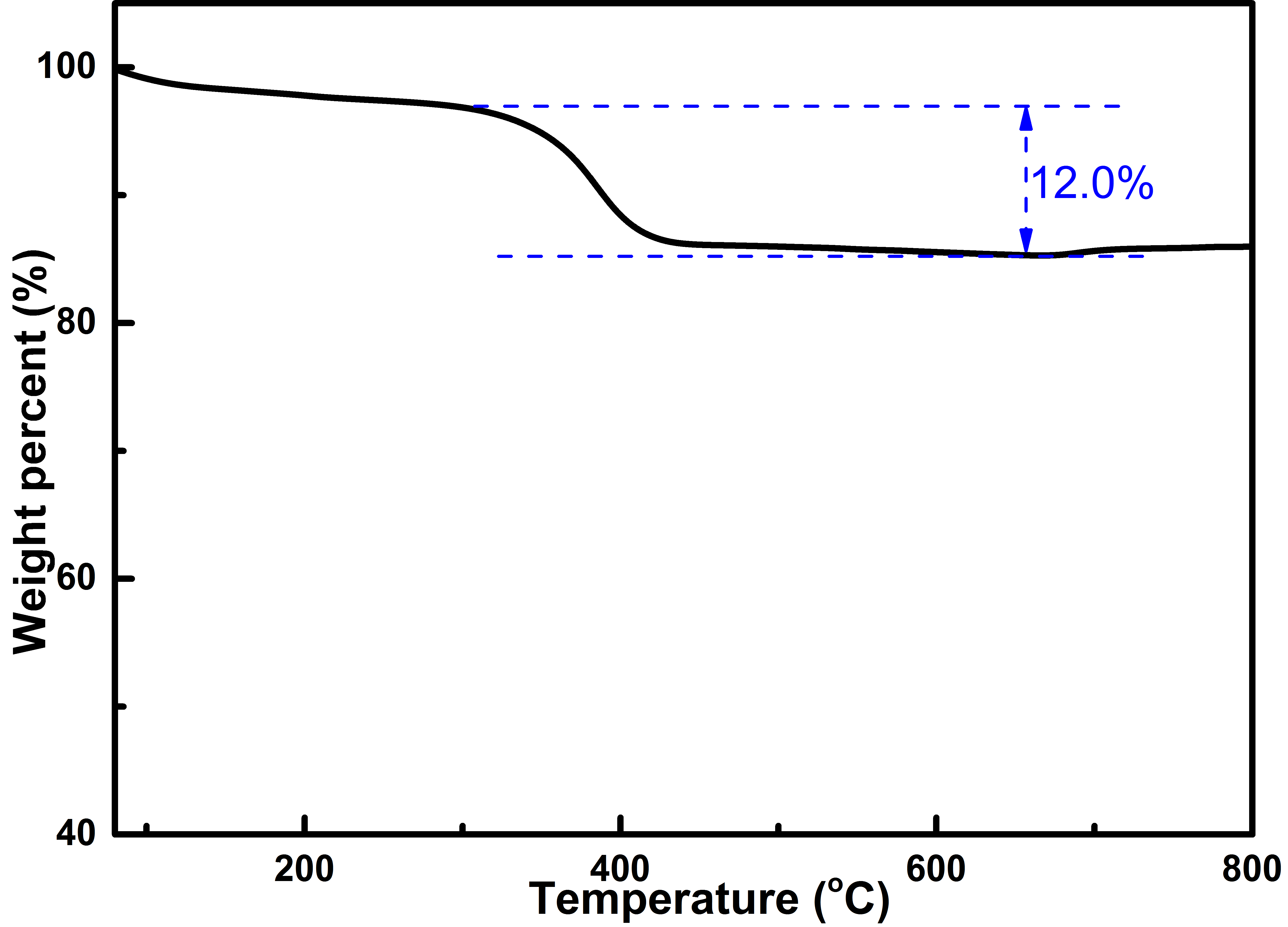


Figure S2: Thermogravimetric analyses of MnFe_2_O_4_-rGO in air.
